# Operator dependency of arterial input function in dynamic contrast-enhanced MRI

**DOI:** 10.1007/s10334-021-00926-z

**Published:** 2021-07-02

**Authors:** Magne Kleppestø, Atle Bjørnerud, Inge Rasmus Groote, Minjae Kim, Jonas Vardal, Christopher Larsson

**Affiliations:** 1 Division of Radiology and Nuclear Medicine, Department of Diagnostic Physics, Oslo University Hospital, Oslo, Norway; 2 Faculty of Medicine, University of Oslo, Oslo, Norway; 3 Unit for Computational Radiology and Artificial Intelligence, Division of Radiology and Nuclear Medicine, Oslo University Hospital, Oslo, Norway; 4 Department of Physics, Faculty of Mathematics and Natural Sciences, University of Oslo, Oslo, Norway; 5 Department of Psychology, Faculty for Social Sciences, University of Oslo, Oslo, Norway; 6 Department of Radiology, Vestfold Hospital Trust, Tønsberg, Norway; 7 Department of Radiology, Asan Medical Center, University of Ulsan College of Medicine, Seoul, Republic of Korea; 8 Department of Radiology and Research Institute of Radiology, University of Ulsan College of Medicine, Asan Medical Center, Seoul, Korea; 9 Department of Radiology, Vestre Viken Hospital Trust, Drammen, Norway; 10 Department of Neurosurgery, Oslo University Hospital, Rikshospitalet, Oslo, Norway

**Keywords:** Dynamic contrast-enhanced MRI, DCE-MRI, Glioblastoma, AIF, Arterial input function, Observer dependency

## Abstract

**Objective:**

To investigate the effect of inter-operator variability in arterial input function (AIF) definition on kinetic parameter estimates (KPEs) from dynamic contrast-enhanced (DCE) MRI in patients with high-grade gliomas.

**Methods:**

The study included 118 DCE series from 23 patients. AIFs were measured by three domain experts (DEs), and a population AIF (pop-AIF) was constructed from the measured AIFs. The DE-AIFs, pop-AIF and AUC-normalized DE-AIFs were used for pharmacokinetic analysis with the extended Tofts model. AIF-dependence of KPEs was assessed by intraclass correlation coefficient (ICC) analysis, and the impact on relative longitudinal change in *K*^trans^ was assessed by Fleiss’ kappa (*κ*).

**Results:**

There was a moderate to substantial agreement (ICC 0.51–0.76) between KPEs when using DE-AIFs, while AUC-normalized AIFs yielded ICC 0.77–0.95 for *K*^trans^, *k*_ep_ and *v*_e_ and ICC 0.70 for *v*_p_. Inclusion of the pop-AIF did not reduce agreement. Agreement in relative longitudinal change in *K*^trans^ was moderate (*κ* = 0.591) using DE-AIFs, while AUC-normalized AIFs gave substantial (*κ* = 0.809) agreement.

**Discussion:**

AUC-normalized AIFs can reduce the variation in kinetic parameter results originating from operator input. The pop-AIF presented in this work may be applied in absence of a satisfactory measurement.

## Introduction

Dynamic contrast-enhanced (DCE) magnetic resonance imaging (MRI) is increasingly being used to assess microvascular properties of tissue in oncology and has proven well suited to quantify brain tumor hemodynamics and damage to the blood–brain barrier. DCE-MRI is a technique in which a series of *T*_1_-weighted images is rapidly acquired before, during and after the administration of a paramagnetic contrast agent (CA) [1]. Using pharmacokinetic (PK) modelling, these dynamic images allow for the quantification of kinetic biomarkers that are of use in the evaluation of tumors. The most commonly used PK model in high-grade glioma (HGG) diagnostics is the extended Tofts model [2], which describes the tissue by the rate constants *K*^trans^ and *k*_ep_ as well as the volume fractions *v*_e_ and *v*_p_.

Quantitative descriptors of tumor vasculature that—in theory—can be obtained independently of equipment and technique, would be a very powerful tool in disease management and monitoring. However, DCE-MRI suffers from a lack of reproducibility across different imaging sites. This is due to several potential sources of error, including inadequate temporal resolution, insufficient acquisition time frame [3], pre-contrast T_1_ measurement uncertainty [4, 5] and difficulty in determining the dynamic CA concentration in plasma (*C*_p_)—the arterial input function (AIF) [6, 7].

Different approaches exist for determining the AIF, including fully automatic algorithms [8], semi-automatic methods [9], manual selection and the use of standard models [10]. It is generally accepted that the AIF is dependent on cardiac output, blood pressure and vascular auto-regulation in the region-of-interest []. The gold standard for DCE-MRI imaging is thus an individual AIF from each time-point in each patient measured in a feeding artery of the pathology/region-of-interest (ROI). The accuracy of this AIF varies by the temporal resolution, potential partial volume effects from low spatial resolution [11] and inter-observer variability from manual selection.

The aim of this study was to investigate the inter-observer variability, among domain experts (DEs), in AIF determination and corresponding variability in kinetic parameters obtained using the extended Tofts model in HGG patients. Further, we define a parametric form of a population-averaged AIF from brain data according to the framework previously published [10].

## Methods

### Patients

Study approval was obtained from the regional medical ethics committee and patients were included only if written informed consent was signed. A total of 118 DCE-MRI examinations from 23 patients (17 males, mean age 53.7 years, range 32–66 years) with histologically confirmed HGG (one grade III and 22 grade IV) were included in a prospective study of early detection of perfusion changes during radiochemotherapy [12]. Patients were imaged once before, thrice during, and up to five times for a maximum of 15 months after initiation of standard treatment regime [13]. In the present work, the initial six imaging time-points in each patient, encompassing two post-radiochemotherapy follow-ups at two and 14 weeks, were considered for inclusion. Examinations were excluded if there were no contrast-enhancing voxels (*N* = 2) or if DCE-MRI was not successful (*N* = 18), resulting in a total of 118 included examinations. A surgical debulking procedure was performed in 22 patients prior to baseline imaging, whilst the remainder received biopsy.

### MRI

All imaging was performed at 3.0 T (Philips Achieva, Philips Medical Systems, Best, The Netherlands), using an eight-channel head coil. DCE images were acquired from a 3D- saturation recovery-based gradient echo sequence. The sequence was designed to minimize water exchange effects by employing a short saturation recovery delay (TD) [14]. Eleven slices covering the tumor volume were acquired with the following key sequence parameters: field of view (FoV): 240 × 240 mm^2^; acquisition matrix: 120 × 120; partial Fourier: 5/8; voxel size: 1.9 × 1.9 × 4 mm^3^; pixel bandwidth: 434 Hz; echo time (TE)/flip angle: 2.5 ms/26°; sensitivity encoding (SENSE) factor: 2.3 in the anterior–posterior phase-encoding direction. Two variants of the DCE sequence were used: one using repetition time (TR)/TD/temporal resolution: 8.2 ms/80 ms/3.4 s, 100 dynamic scans and scan time: 5:40 (variant 1) in seven patients for a total of 38 examinations, and one using TR/TD/temporal resolution: 5.1 ms/50 ms/2.1 s, 150 dynamic scans and scan time: 5:10 (variant 2) in 16 patients for a total of 80 examinations. 0.1 mmol/kg gadobutrol (Gadovist^®^, Bayer Schering Pharma AG, Berlin, Germany) was administered after a pre-contrast baseline duration of five timepoints using a power injector at a rate of 3 ml/s, immediately followed by a 20 ml saline flush at the same rate. The full imaging protocol included the following structural image series: sagittal 3D fluid-attenuated inversion recovery (FLAIR) images, axial 2D *T*_2_-weighted images and sagittal 3D *T*_1_-weighted gradient echo images acquired before and after CA administration. All image series were acquired before the DCE series except the CA-enhanced *T*_1_-weighted images which were acquired directly afterwards. Tumor region-of-interest (ROI) containing contrast-enhancing tissue was defined in all examinations by a radiologist (4 years of experience) using a previously described method [12, 15].

### AIF extraction

**Fig. 1:**
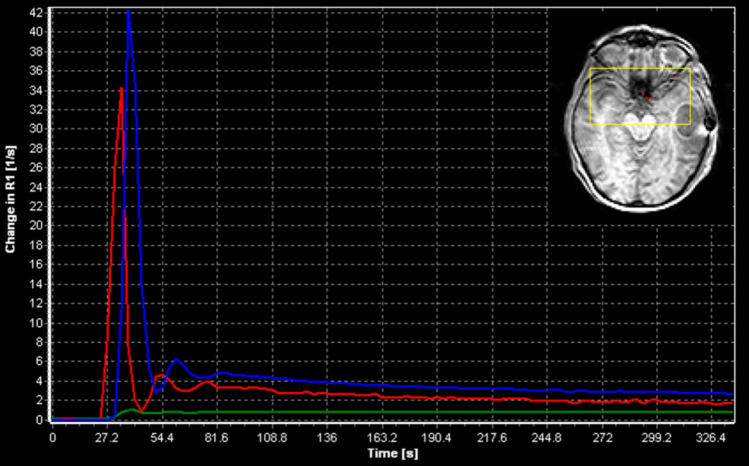
Outline of the arterial input function (AIF) extraction process. Top right: the search area (yellow rectangle) is placed in an area likely to contain arterial signal, and the algorithm selects the best pixels (red squares). The AIF is the mean signal of the selected pixels and is plotted in red. The venous output function is detected similarly and is plotted in blue along with mean tumor

AIFs were determined independently in each DCE series by three DEs (two radiologists with 1 (J. V.) and 5 (M. Kim.) years of experience and one clinician with 10 years of experience in DCE-MRI (C. L.)) using a semi-automatic algorithm based on automated identification of most likely AIF voxels using K-means clustering from a user-defined search region, implemented in the software tool nordicICE (NordicNeuroLab, Bergen, Norway) [8]. The AIF search region was selected independently by each DE to include the large intracerebral arteries according to the tumor location and hence the available volume covered by the DCE acquisition. Since the K-means method is iterative with a random seed starting point, repeated AIF searches may not result in identical results. Hence, multiple AIF searches were performed for a selected search region and the AIF with the highest first-pass arterial signal peak and the highest signal tail during the wash-out phase was chosen. This process was repeated until the AIF was considered satisfactory by the DE. An overview of the process is given in Fig. 1. In addition, a venous output function from the large sinuses was obtained in each examination for AIF partial volume correction [3].

The AIFs were supplied as single-column text files containing change in longitudinal relaxation rate $\Delta {R}_{1}={R}_{1}\left(t\right)-{R}_{1}\left(0\right)$ from baseline for each timepoint *t*, which was converted to CA concentration by:

1 $$\begin{array}{c}{C}_{\mathrm{p}}\left(t\right)= \frac{{R}_{1}\left(t\right)-{R}_{1}\left(0\right)}{{r}_{1}\left(1-\mathrm{Hct}\right)},\end{array}$$

where *r*_1_ = 4.4 mM^−1^ s^−1^ is the relaxivity of gadobutrol at 3.0 T [16] and Hct is the hematocrit, set to 0.45. *R*_1_(0) of arterial blood at 3.0 T was assumed to be 0.60 s^−1^ [16].

This procedure resulted in a total of 354 DE-defined AIFs from 23 patients, of which 114 AIFs from seven patients were from DCE sequence variant 1 and the remaining 240 AIFs in 16 patients were from DCE sequence variant 2.

### Population-averaged AIF

A population-averaged AIF (pop-AIF) was created by fitting the mean of all measured AIFs with DCE sequence variant 2 (*N* = 240 AIFs) to the sum of two Gaussians and an exponential modulated by a sigmoid function, as described by Parker et al. [10]:

2 $$\begin{array}{*{20}c} {C_{{\text{b}}} \left( t \right) = \sum\limits_{{n = 1}}^{2} {\frac{{A_{n} }}{{\sigma _{n} \sqrt {2\pi } }}} {\text{exp}}\left( {{\raise0.7ex\hbox{${ - \left( {t - T_{n} } \right)^{2} }$} \!\mathord{\left/ {\vphantom {{ - \left( {t - T_{n} } \right)^{2} } {2\sigma _{n}^{2} }}}\right.\kern-\nulldelimiterspace} \!\lower0.7ex\hbox{${2\sigma _{n}^{2} }$}}} \right) + \frac{{\alpha \;{\text{exp}}\left( { - \beta t} \right)}}{{1 + {\text{exp}}\left( { - s\left( {t - \tau } \right)} \right)}}} \\ \end{array} .$$

Here, *A*_*n*_ are scaling factors, *T*_*n*_ are centers and *σ*_*n*_ are widths of the *n*th Gaussian; *α* and *β* describe the amplitude and slope of the exponential function, respectively, and *s* and *τ* are the width and center of the sigmoid. Prior to calculating the mean AIF, each individual AIF was time-shifted so that the peak occurred at the same timepoint.

### Pharmacokinetic modeling

The DCE time series were analyzed using the extended Tofts model according to:

3 $$\begin{array}{c}{C}_{\mathrm{t}}\left(t\right)={K}^{\mathrm{trans}}{\int }_{0}^{t}{C}_{\mathrm{p}}\left(\tau \right)\mathrm{exp}\left(-\frac{{K}^{\mathrm{trans}}\left(t-\tau \right)}{{v}_{\mathrm{e}}}\right)\mathrm{d}\tau +{v}_{\mathrm{p}}{C}_{\mathrm{p}}\left(t\right) ,\end{array}$$

where *C*_t_(*t*) is the time-dependent CA concentration in tissue, *C*_p_(*t*) is the time-dependent CA concentration in blood plasma (the AIF), *K*^trans^ is the flux of contrast agent from the intravascular space (IVS) to interstitium, *k*_ep_ is the flux of contrast agent back to the IVS, *v*_e_ is the ratio of *K*^trans^ and *k*_ep_, denoting the fraction of extracellular, extravascular volume and *v*_p_ is the fractional plasma volume.

From Eq. 3, it is evident that errors in the amplitude of ${C}_{\mathrm{p}}(t)$ will directly scale to corresponding errors in *K*^trans^ and *v*_p_. Therefore, to assess the contribution of differences in AIF peak values, the analysis was repeated using AIFs normalized to an area under the curve (AUC) equal to the mean of all measured AIFs (600 mM s). Because each patient received the same CA dose at each imaging session, the AIF AUC is expected to remain constant, and normalization should therefore reduce variability in parameter estimates due to errors in measured peak AIF amplitudes, but at the cost of loss of absolute quantitative parameter estimates. Consequently, kinetic analysis was performed with seven different AIFs in each examination: three DE-supplied, the same three AUC-normalized and the pop-AIF obtained by fitting the mean AIF across all examinations to Eq. 2. From each analysis, the median parameter values from each tumor ROI were extracted and used for comparison. Voxels in which parameters *v*_e_ or *v*_p_ took an unphysiological value outside the range 0–100% was excluded from median calculation for all AIFs in that examination. The PK modelling and AIF analysis were performed in MATLAB 2020a (MathWorks, Natick, MA, United States).

### Statistical analysis

AIF peak and AUC, as well as estimates of parameters *K*^trans^, *k*_ep_, *v*_e_ and *v*_p_, were used for statistical analysis. The median value of each parameter in each tumor ROI was used for analysis. AIF characteristics were assessed using Wilcoxon signed-rank test on the AIF peak and AUC of each DE-supplied AIF against each other (e.g. DE 1 vs DE 2; four combinations). Intraclass correlation coefficient (ICC) estimates with 95% confidence intervals (CI) were calculated based on an absolute-agreement, two-way random-effects model [17]. The ICC estimate evaluates the degree of inter-operator agreement in the KPE estimates within each examination, such that identical results from all AIFs in every examination would give ICC = 1. ICCs were estimated both using the complete dataset, and including only the examinations with DCE sequence variant 1 (*N* = 38) to assess the performance of the pop-AIF. To investigate the effect of different AIFs on predicting clinical outcome, the change in median *K*^trans^ for each patient from the first to the sixth imaging time-point was compared using the measured DE-AIFs and the normalized variants. An increase or decrease of more than 10% was labeled as, respectively, progression or remission, and the scores were assessed with Fleiss’ kappa using linear weights. The kappa statistic was interpreted according to the Landis and Koch benchmarking scale [18]. ICC and Fleiss’ kappa statistics were performed using Stata SE 16.1 (StataCorp LLC, College Station, TX, United States) with the kappa, etc., command [19].

## Results

### Population-averaged AIF

**Fig. 2:**
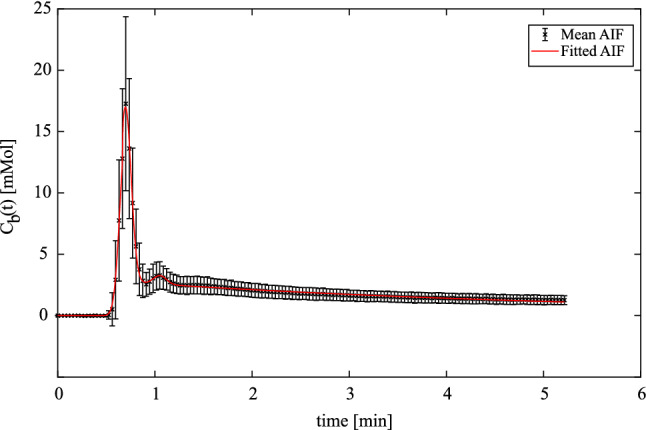
Mean arterial input function (AIF) across all domain expert-measured AIFs in individual subjects at all timepoints represented with crosses, and error bars showing ± 1 standard deviation. The fitted population AIF is shown in red

**Fig. 3:**
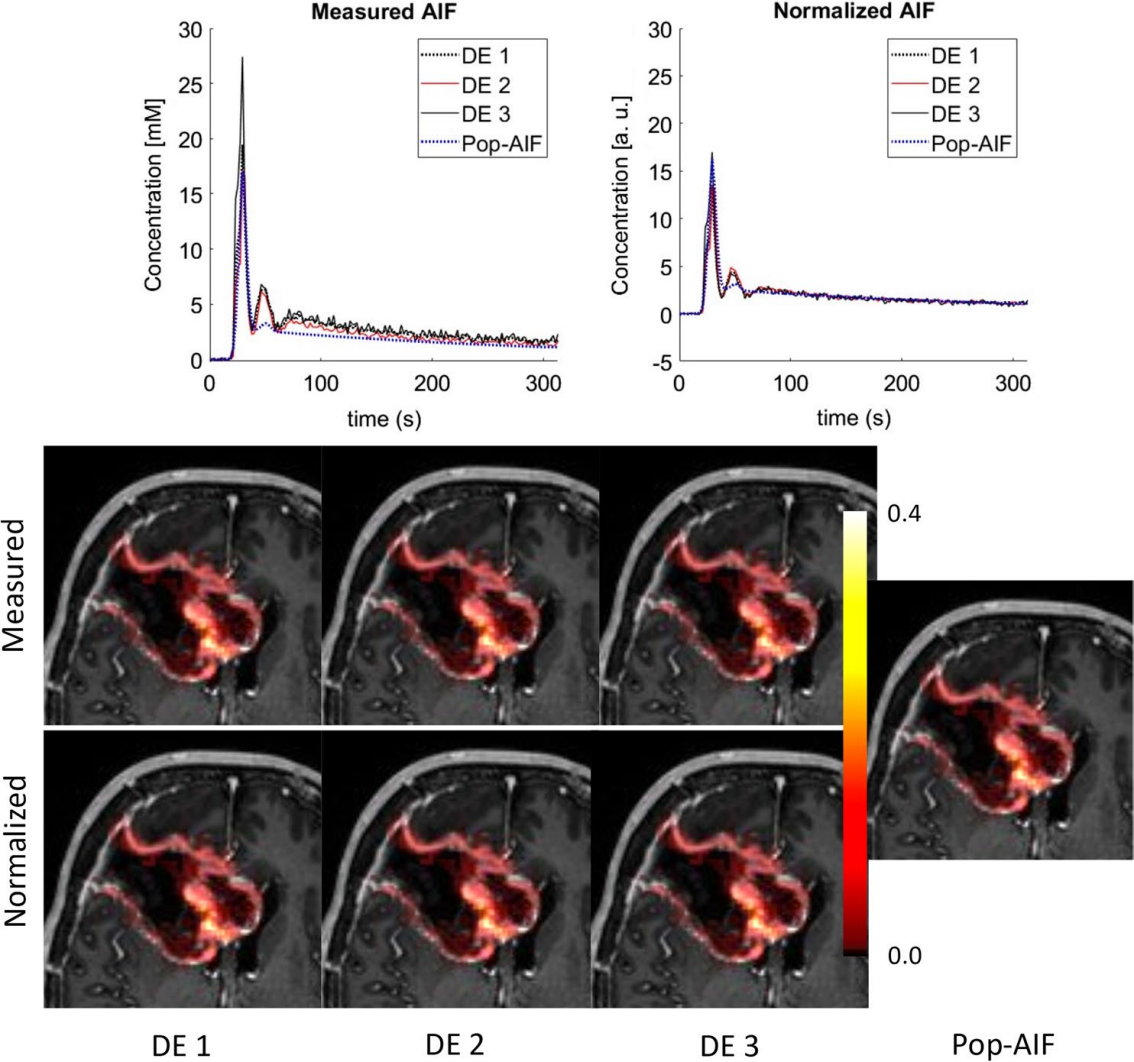
Top: measured (left) and normalized (right) AIFs from each domain expert (DE) in a sample examination, with the time-aligned population AIF (pop-AIF) (11). Bottom: resulting tumor *K*^trans^ maps overlaid on structural post-contrast T1 images. The *K*^trans^ color scale ranges from 0.0 to 0.4 min^−1^. Median *K*^trans^ of the whole tumor for DE 1, 2 and 3 and pop-AIF was, respectively: 0.049 min^−1^, 0.053 min^−1^, 0.044 min^−1^, 0.101 min^−1^ (measured AIFs) and 0.071 min^−1^, 0.067 min^−1^, 0.071 min^−1^ and 0.080 min^−1^ (normalized AIFs)

Figure 2 shows the fitted population AIF together with the mean *C*_b_(*t*). The fitted value for each AIF parameter (Eq. 2) is given in Table 1. The fitted AIF is seen to closely follow the mean AIF, with a first-pass peak followed by a smaller second-pass peak and subsequent washout. The standard deviation of the mean AIF demonstrates a higher variation in measured AIFs during the early phase than during washout. A comparison of the pop-AIF and DE-AIFs with resulting *K*^trans^ maps is shown in Fig. 3.

**Table 1:** Parameter values and their standard deviation (SD) for the fitted population AIF (Eq. 2)

Parameter	*A*_1_	*A*_2_	*T*_1_	*T*_2_	*σ*_1_	*σ*_2_	*α*	*β*	*s*	*τ*
Value	2.092	0.118	0.696	1.051	0.059	0.067	3.161	0.200	818.4	0.667
SD	0.028	0.032	0.001	0.016	0.0008	0.0178	0.0982	0.011	0.000	0.001
Units	mmol min	min	min	min	min	min	mmol	min^−1^	min^−1^	min

### Statistical analysis

The AIF peaks were found to be significantly different between all domain experts (*p* = 0.03), while AUC was found to be different between DE 2 and 3 (*p* < 10^–8^), but not between 1 and 2 (*p* > 0.80) or 1 and 3 (*p* > 0.14).

Table 2 shows ICC for median kinetic parameter values from the 118 examinations. There is a moderate to substantial agreement for all kinetic parameters when using the measured AIFs [18]. Inclusion of the pop-AIF yields a lower agreement estimate for *k*_ep_, while the other parameters are negligibly affected. The normalized AIFs give an almost perfect (0.8–1.00) agreement for parameters *K*^trans^ and *v*_e_, while *v*_p_ ‘s agreement is substantial (0.61–0.80). As expected, *k*_ep_ is the least affected by normalization of the AIF, as it is not sensitive to the AIF amplitude [20]. Table 3 shows the same ICC estimates calculated from only the examinations using DCE sequence variant 1 and demonstrates a similar relationship between ICC estimates with and without inclusion of the pop-AIF.

**Table 2:** Intraclass correlation coefficient (95% confidence interval) of median parameter values estimated using measured and normalized arterial input functions (AIF) from domain experts (DE) 1–3 and population AIF (pop-AIF), including all imaging time-points in all patients (*N* = 118)

AIFs used	*K*^trans^	*k*_ep_	*v*_e_	*v*_p_
DE 1–3	0.670 (0.543–0.766)	0.762 (0.694–0.820)	0.702 (0.588–0.787)	0.510 (0.395–0.617)
DE 1–3 and pop-AIF	0.671 (0.572–0.754)	0.692 (0.619–0.760)	0.678 (0.581–0.758)	0.497 (0.399–0.594)
AUC-normalized DE 1–3	0.951 (0.934–0.964)	0.765 (0.698–0.823)	0.891 (0.857–0.921)	0.698 (0.616–0.769)
AUC-normalized DE 1–3 and pop-AIF	0.940 (0.921–0.956)	0.694 (0.622–0.761)	0.858 (0.817–0.892)	0.693 (0.620–0.760)

**Table 3:** Intraclass correlation coefficient (95% confidence interval) of median parameter values estimated using measured and normalized arterial input functions (AIF) from domain experts (DE) 1–3 and population AIF (pop-AIF), including only examinations with DCE sequence variant 1 (*N* = 38)

AIFs used	*K*^trans^	*k*_ep_	*v*_e_	*v*_p_
DE 1–3	0.559 (0.364–0.725)	0.746 (0.611–0.850)	0.697 (0.507–0.823)	0.446 (0.250–0.635)
DE 1–3 and pop-AIF	0.552 (0.379–0.710)	0.761 (0.645–0.853)	0.668 (0.476–0.805)	0.439 (0.275–0.614)
AUC-normalized DE 1–3	0.967 (0.950–0.984)	0.758 (0.623–0.855)	0.928 (0.879–0.953)	0.792 (0.675–0.879)
AUC-normalized DE 1–3 and pop-AIF	0.965 (0.944–0.980)	0.765 (0.650–0.856)	0.923 (0.876–0.955)	0.803 (0.703–0.882)

**Fig. 4:**
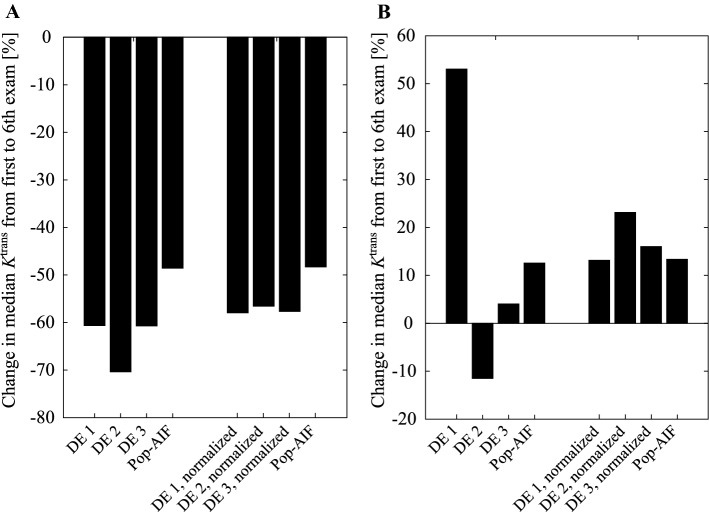
Relative change in *K*^trans^ in two sample patients from the first to the sixth exam estimated using AIFs from each domain expert (DE) and population AIF (pop-AIF) (11). **a** Patient with similar development in *K*^trans^ with all DE-measured and population AIF. The largest absolute change with measured AIFs was − 0.016 min^−1^ (DE 2), and 0.0091 min^−1^ (DE 1) with normalized AIFs. **b** A patient where measured AIFs from the three DEs give diverging progression, while the normalized AIFs give a unidirectional progression. With measured AIFs from DE 1 the change is + 0.022 min^−1^, while normalized AIFs from DE 2 gives a change of − 0.014 min^−1^

Figure 4 shows the relative *K*^trans^ change from baseline to the sixth exam for two sample patients that demonstrate different degrees of observer dependence. Panel B represents a worst-case scenario, where different DE-AIFs produce opposing estimates of disease progression.

When change in median *K*^trans^ is segmented into remission (< 10%), *status quo* (− 10% ≤ and ≤  + 10%) and progression (> + 10%), Fleiss’ kappa (3 raters, 23 subjects) is calculated as 0.595 with a 95% CI of (0.363, 0.827) for the measured AIFs, suggesting a fair to moderate agreement, while the normalized counterparts yield a kappa of 0.809 with CI (0.661, 0.958), suggesting a substantial to almost perfect agreement.

## Discussion

The aim of the present work was to investigate the effect of differences in user-defined AIFs on kinetic parameters estimated from DCE using the extended Tofts model on data from HGG patients. Three experienced DEs individually produced AIFs for 118 examinations of 23 patients with HGG, following a common guideline that included the use of a semi-automatic detection algorithm. Additionally, a population-averaged AIF was fitted to a subset of the measured AIFs. A fixed *T*_1_(0) both in tissue and blood was assumed in all patients, and all analyses were performed at the same facility without input from the DEs other than their supplied AIFs. Therefore, the results of the kinetic analyses in this study should not be influenced significantly by factors other than the difference in AIFs.

The results in this study show moderate to substantial agreement in kinetic parameters estimated with AIFs from different DEs (Table 2). Agreement in estimated parameters is not substantially affected when the pop-AIF is included. This suggests that the pop-AIF represents an appropriate alternative to manually measured AIFs on a per-examination basis. The AUC-normalized DE-AIFs is seen to yield higher agreement in parameter estimates for *K*^trans^, *v*_e_ and *v*_p_, as expected from the known sensitivity of these parameters to the AIF peak amplitude. The parameter *k*_ep_ depends more on the shape of the AIF [20] and is therefore virtually unaffected by the AIF normalization. This is in agreement with previous work investigating the effects of measuring the AIF for brain DCE in different vessels [21], which found a strong correlation between lower AIF peak and overestimation of all parameters except *k*_ep_.

Comparing the relative change in parameter estimates over time as an indicator for disease progression shows that the use of AUC-normalized AIFs increases the agreement between DEs. The case presented in Fig. 4b represents a worst-case scenario where longitudinal change in *K*^trans^ is labeled differently (progression, stable, remission) by each of the DE-AIFs, while all AUC-normalized DE-AIFs and the pop-AIF indicate progression. This demonstrates that a single DCE series can contain several clusters of voxels that produce subjectively acceptable, yet consequentially different AIFs. This operator-dependency is reduced with the AUC-normalized DE-AIFs and completely removed with the pop-AIF, at the cost of potential loss of relevant information.

The AIFs in this study were not accompanied by per-examination measurement of hematocrit, which is known to fluctuate significantly during chemotherapy treatment [22]. Hematocrit variations would lead to a true variation in AIF peak amplitude, which would not be reflected in the AUC-normalized AIF or the pop-AIF. However, if hematocrit values were available, this could be incorporated as an additional per-patient and per-examination scaling factor in the resulting AUC-normalized or population-based AIFs [23].

This study included data acquired with two slightly different DCE-MRI sequences, differing in TR, TE, TD and number of dynamic scans. The pop-AIF was derived using only data from the sequence variant with the largest number (variant 2, *N* = 220) of scans. To confirm that this pop-AIF was valid for both sequence variants, the ICC was compared including either all data from both sequences and only data from sequence variant 1. The results (Tables 2, 3) show that agreement is equally affected when including the pop-AIF in both datasets, indicating that the pop-AIF can be used on data acquired with sequence parameters deviating from the ones used when defining the pop-AIF.

One major challenge with the DCE-MRI field is lack of standardization between sites and studies. Although white papers suggesting standards for MRI protocol and analysis exist [2], there is still a substantial variation in both acquisition and analysis methods in the published data, making comparison of quantitative parameter estimates challenging. In treatment response studies, like the one presented here, the absolute values of the kinetic parameters may be of less importance and the emphasis should be on the ability of a given method to accurately detect parameter changes during the course of disease progression and treatment response. This is in line with previous findings in a large multicenter study concerning prostate cancer [7]. Our results suggest that this can readily be achieved through a combination of standardized and fixed protocols for AIF determination and a fixed imaging protocol over time. Further, and more importantly, our results suggest that the derivation of a population-wide AIF derived from the mean of many DCE-MRI examinations acquired on the same system provides a good alternative to the need for time-consuming identifications of individual AIFs by domain experts. It is, however, important to stress that such population AIF may need to be adjusted according to site-specific variations in MRI protocol and scanner type. Still, the population AIF obtained here, based on the same functional form as previously suggested by Parker for abdominal use [10], may form the basis for a similar pop-AIF for use in brain DCE-MRI applications.

In conclusion, normalizing the measured AIFs to a reasonable AUC can serve to reduce the variation in kinetic parameter results that stem from operator input. Further, using a parametric population AIF for the brain as presented in this study may be applied in absence of a satisfactory measurement in kinetic parameter estimation from DCE-MRI data in HGG patients.

## Abbreviations

AIF: Arterial input function
AUC: Area under the curve
CA: Contrast agent
DCE: Dynamic contrast-enhanced
DE: Domain expert
FLAIR: Fluid-attenuated inversion recovery
HGG: High-grade glioma
IVS: Intravascular space
KPE: Kinetic parameter estimate
MRI: Magnetic resonance imaging
PK: Pharmacokinetic
Pop-AIF: Population-averaged AIF
ROI: Region-of-interest
TD: Saturation recovery delay
TE: Echo time
TR: Repetition time
